# Biomaterials for Local, Controlled Drug Delivery to the Injured Spinal Cord

**DOI:** 10.3389/fphar.2017.00245

**Published:** 2017-05-10

**Authors:** Alexis M. Ziemba, Ryan J. Gilbert

**Affiliations:** Department of Biomedical Engineering and Center for Biotechnology and Interdisciplinary Sciences, Rensselaer Polytechnic Institute, TroyNY, USA

**Keywords:** spinal cord injury, inflammation, regeneration, drug delivery, biomaterials, hydrogels, particles, fibers

## Abstract

Affecting approximately 17,000 new people each year, spinal cord injury (SCI) is a devastating injury that leads to permanent paraplegia or tetraplegia. Current pharmacological approaches are limited in their ability to ameliorate this injury pathophysiology, as many are not delivered locally, for a sustained duration, or at the correct injury time point. With this review, we aim to communicate the importance of combinatorial biomaterial and pharmacological approaches that target certain aspects of the dynamically changing pathophysiology of SCI. After reviewing the pathophysiology timeline, we present experimental biomaterial approaches to provide local sustained doses of drug. In this review, we present studies using a variety of biomaterials, including hydrogels, particles, and fibers/conduits for drug delivery. Subsequently, we discuss how each may be manipulated to optimize drug release during a specific time frame following SCI. Developing polymer biomaterials that can effectively release drug to target specific aspects of SCI pathophysiology will result in more efficacious approaches leading to better regeneration and recovery following SCI.

## Introduction

Affecting approximately 17,000 new people in the United States (US) each year, SCI is devastating because there is currently no cure, and individuals with SCI experience permanent paraplegia or tetraplegia (NSCISC, 2016). Following SCI, vertebrae surrounding the soft spinal cord tissue are dislodged, resulting in the compression of the spinal cord and death of glia and neurons. This initial mechanical damage propagates away from the epicenter of the injury site via secondary injury, preventing regeneration of spinal cord tissue. The aim of this review is to discuss experimental approaches utilizing biomaterials to deliver drugs locally to the injured spinal cord. We then seek to communicate that careful selection of biomaterials and therapeutic release strategies is necessary to target the dynamically changing physiological response following SCI.

## Current Pharmaceutical Treatments for SCI

Clinical treatment of SCI focuses on pain and spasticity management. Musculoskeletal and neuropathic pain are often addressed with anti-depressants, analgesics, and anti-convulsants, but patient palliation is minimal (Cardenas and Jensen, 2006; Teasell et al., 2010; Rabchevsky and Kitzman, 2011; Rabchevsky et al., 2011). Unfortunately, alleviation of these symptoms using pharmaceuticals does nothing to regenerate axons or restore lost function. Several recent or current clinical trials utilize pharmaceuticals to reduce inflammation with the hope of improving functional outcomes. Currently, three pharmaceuticals have been studied or are being studied in the clinic, including MP, minocycline, and EPO (Singh et al., 2012; Kabu et al., 2015). In this section, experimental studies are presented that suggest the mechanism by which these molecules benefit the injured spinal cord. Further, results of clinical trials are shared to disclose the current challenges in applying these therapies to individuals with SCI.

### Pharmacological Agents Tested within Clinical Trials

Methylprednisolone is the most commonly studied SCI therapeutic and has been examined in many preclinical studies. MP is a corticosteroid capable of dampening multiple inflammatory pathways (Hsu and Dimitrijevic, 1990). For example, intravenous MP treatment in rats with T nine level (T9) spinal cord injuries resulted in fewer neutrophils and macrophages at the lesion site (Bartholdi and Schwab, 1995). MP also reduces pro-inflammatory cytokine levels. TNF-α is a major cytokine involved in the acute pro-inflammatory response, and nuclear factor κ-B (NF-κB) is a transcription factor activated by TNF-α (Lawrence, 2009). MP reduced TNF-α levels and NF-κB binding in a rat T9-10 contusion injury model when delivered intravenously (Xu et al., 1998). In addition to reducing levels of pro-inflammatory factors, MP increases neurotrophic factor production and cytokine modulation. MP administration increased the level of NGF in the lesion of rats with a T9 contusion injury (Fumagalli et al., 2008).

As described above, several experimental MP approaches dampen secondary injury responses; however, other studies demonstrated no effect on secondary injury factors following MP administration. As an example, a preclinical study involving pigs with contusion injury showed no change in levels of prostaglandin E2 (lipid involved in inflammation), glutamate (excitatory neurotransmitter), and citrulline (byproduct in synthesis of nitric oxide, which is present under oxidative stress) when MP was delivered intravenously or intrathecally (Bernards and Akers, 2006). Despite preclinical evidence showing mixed efficacy of MP in animal models of SCI, MP is approved by the FDA for use in acutely injured patients. MP delivered via bolus intravenous infusion within the first 8 h post-injury enabled sensory and motor improvement in SCI patients (Bracken et al., 1990). While approved, there is ongoing debate on the therapeutic benefit of MP clinically, as the benefit of MP use does not always outweigh the side effects. Some side effects include increased risk of general infection, increased risk of respiratory infection, and hyperglycemia (Bydon et al., 2014).

Minocycline, a tetracycline antibiotic, was also extensively studied in preclinical SCI models and clinical trials. In preclinical studies, intravenous minocycline reduced mortality and improved BBB motor recovery scores in a mouse T3-4 compression injury model (Wells et al., 2003). Similarly, intraperitoneal injection of minocycline in a rat T9-10 contusion injury reduced caspase-3 activity (involved in cell apoptosis), diminished lesion volume, and ultimately improved BBB scores 24 days post-injury (Lee et al., 2003). Intraperitoneal minocycline delivered to rats with a T9 contusion injury prevented increased cytochrome c levels following injury while also improving BBB scores at weeks 3 and later (Teng et al., 2004). Unfortunately, benefits observed within preclinical models did not translate to improved functional outcomes in human clinical trials. A study administering intravenous minocycline within 12 h post-injury then twice daily demonstrated insignificant improvements in the ASIA neurological assessment (Casha et al., 2012).

Erythropoietin is a growth factor with neuroprotective effects when administered following SCI (Singh et al., 2012). Within preclinical testing, intraperitoneal injection of EPO 30 min post-T9 contusion injury in rats resulted in higher BBB functional scores than in rats that received no drug or treatment with MP. At 3 days post-injury, a cohort of animals in the same study showed higher levels of NGF mRNA proximal to the lesion epicenter following EPO exposure (Fumagalli et al., 2008). Rats with T9-11 compressive injury injected intraperitoneally with EPO 30–60 min post-injury had increased levels of glutathione and decreased levels of TNF-α, suggesting improved management of oxidative stress and inflammation (Yazihan et al., 2008). A recently completed clinical trial revealed that some patients benefit from intravenous EPO treatment. In a clinical trial, 27% of patients that received intravenous EPO saw a reduction in ASIA grade compared to 0% treated with MP (Costa et al., 2015).

Despite experimental studies demonstrating secondary injury benefits, none of the above agents are sufficient to enable complete restoration to a healthy spinal cord. Administering drugs to the spinal cord is complex, with many injury phenomena requiring treatment. Furthermore, it is a challenge to provide a local, sustained release of certain drugs to target these injury phenomena. The following section discusses reasons for diminished efficacy of drugs administered to the spinal cord.

### Reduced Efficacy of Pharmaceuticals Tested in Clinical Trial – A Need for Advanced Materials to Deliver Pharmaceuticals Locally

While many pharmaceuticals are tested within experimental models and some in clinical trials, failure of these pharmaceuticals to appreciably improve functional outcomes clinically is a result of several deficiencies. Clinically, a common strategy to administer a drug is using a bolus injection of drug followed by a continuous infusion. This strategy is used for intravenous delivery of MP (Braughler et al., 1987; Bracken et al., 1990). The systemic delivery of MP requires a higher dose to ensure a therapeutic concentration at the injury site. Furthermore, such high doses of MP systemically result in a compromised immune system, pneumonia, and myopathy (Gerndt et al., 1997; Qian et al., 2004).

Many of the cellular responses to SCI occur over longer time frames [e.g., macrophages are present days to months (Fleming et al., 2006)], and some therapies have short half-lives, such as growth factors and enzymes (Liu et al., 2013). Thus, clinicians must administer these drugs continuously to maintain therapeutic doses at the injury site. Extended delivery is also important when delivering molecules such as antisense oligonucleotides that can alter gene expression by knocking down specific genes (DeVos and Miller, 2013). Since genetic knockdown through the use of oligonucleotides is transient, the modification may be of insufficient duration to significantly improve SCI. To guarantee a continuous, long-term delivery at the lesion site, mini-osmotic pumps are used to administer drugs intrathecally over an extended period of time and can remedy the short lifetime of bolus injections. However, mini-osmotic pumps increase the risk of infection and are unable to consistently deliver therapeutic concentrations to injured tissue due to drug diffusion out of the injury site (Penn et al., 1995; Loubser and Akman, 1996; Bottros and Christo, 2014).

Once the SCI stabilizes and the blood-brain barrier reforms, many therapies initially able to enter the lesion site due to a compromised blood-brain barrier are no longer able to enter in concentrations sufficient for therapeutic benefit. Typically, drugs with molecular weights larger than 400–500 Da that form more than 8–10 hydrogen bonds with surrounding water molecules are unable to pass through the blood-brain barrier (Pardridge, 2005; Upadhyay and Upadhyay, 2014). To circumvent blood-brain barrier permeability issues, these large, hydrophilic drugs must be delivered locally through an intrathecal route or into the cerebral spinal fluid in the subarachnoid space. In cynomolgus monkeys, intrathecal delivery of idursulfase resulted in higher drug levels in cerebrospinal fluid compared to intravenous administration (Xie et al., 2015). This method of delivery is often used for treatment of pain and spasticity relief in experimental animal studies (Bowersox et al., 1996; Jain, 2000). Unfortunately, this delivery strategy is not sustainable due to repeated invasiveness.

While several therapeutic approaches show promise within experimental injury models, the drawbacks of pharmacological therapies limit their potential use clinically. One solution to counteract the above shortcomings is to use drug-releasing biomaterials. Their local, tunable delivery can prevent detrimental side effects of drugs delivered systemically, such as a compromised immune system. These polymer-based materials are implantable or sometimes injectable. Commonly used biomaterials for the CNS include hydrogels, particles, and fibers/conduits (Straley et al., 2010; Varma et al., 2013; Assunção-Silva et al., 2015). These materials provide a matrix to aid in tissue restoration and are designed to degrade over time. Their morphological properties are easily tuned by changes in chemical composition, which enables modulation of drug release. The following section describes the most common polymers and material types used in the CNS.

## Biomaterials for SCI and Strategies to Tune the Rate of Release from Biomaterials

Depending on the aspect of SCI targeted, the material selection criteria vary. Biomaterials should provide structural support to regenerating axons and glia migrating into the injury site. The biomaterial’s mechanical properties, particularly the stiffness, should be similar to the mechanical characteristics of nervous tissue. Additionally, the biomaterial should degrade and be replaced by regenerating tissue. The materials and their breakdown products should also be non-toxic and elicit minimal immune response. Importantly, the drug release kinetics should be tunable. Hydrogels, particles, and fibrous materials fulfill the criteria mentioned above and will be subsequently reviewed here. Approaches for altering the duration of drug release from these materials will also be discussed.

### Hydrogels as SCI Therapeutics

**FIGURE 1:**
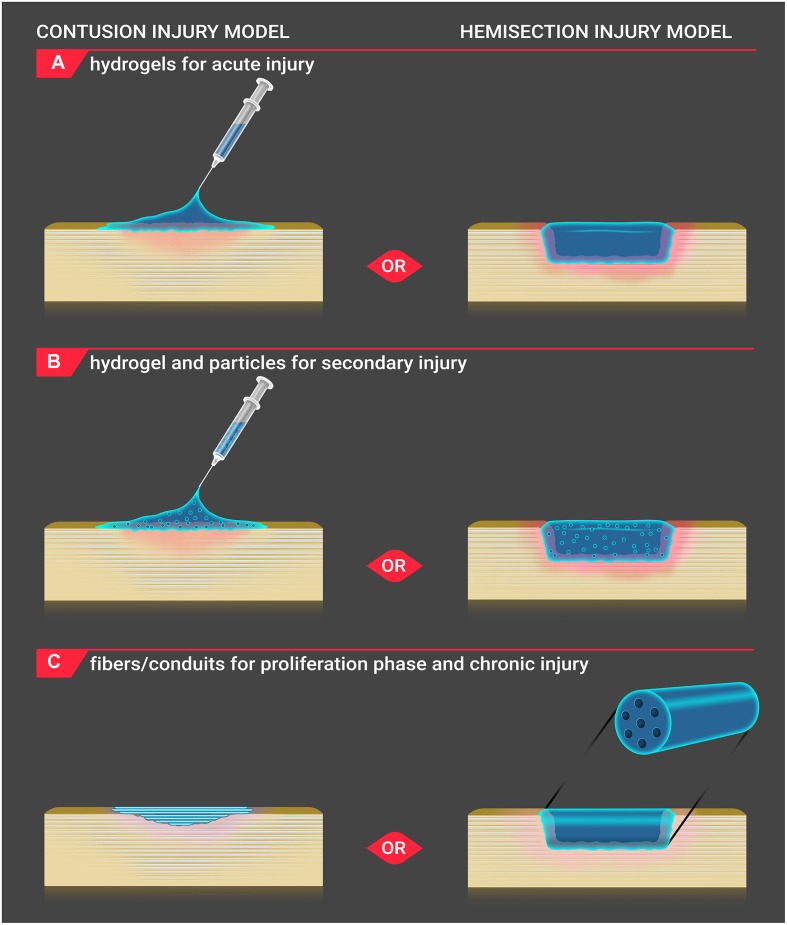
**Biomaterial application to injury site**. Biomaterials are applied to the injury site for both contusion and hemisection SCI models *in vivo*. **(A)** For acute injury, hydrogels are injected onto the contusion injury site, and solidified gels are positioned into the hemisection injury site. **(B)** For secondary injury, hydrogels incorporating particles are injected onto the contusion injury site, and solidified gels containing particles are placed into the hemisection injury site. **(C)** During the proliferation and chronic injury phases, a shallow cavity develops within the lesion of contusion injury. Fibers are positioned below the dura within the contusion injury cavity, and conduit scaffolds are inserted within the hemisection injury to bridge the healthy tissue. As the conduit is a more rigid structure, its use in an irregularlyshaped contusive injury is less applicable.

Hydrogels are biomaterials consisting of hydrophilic polymer networks, and their polymer chain entanglement or crosslink density may be modified to match the mechanical characteristics of the native spinal cord. Several injectable hydrogels are used as drug delivery vehicles because they can be applied to the intrathecal space of the spinal cord. Injectable hydrogels are desirable for contusive SCI, which possesses irregular injury geometries (**Figure 1A**). Hydrogels were first placed into the CNS in the mid-1990s when PHEMA hydrogels containing Schwann cells were implanted into rat lesioned optic tract. Plant et al. saw axons penetrating two thirds of the scaffolds studied (Plant et al., 1995). Similarly, collagen IV was combined with PHEMA and Schwann cells and implanted into the lesioned optic tract, resulting in increased neuron penetration (Plant et al., 1998).

Woerly et al. conducted small craniotomies and placed PHPMA hydrogels functionalized with Arg-Gly-Asp peptides into the cerebrum. Neurofilament-positive processes penetrated the hydrogel, and GFAP-positive glia were supported (Woerly et al., 1995). PHPMA was also functionalized with aminosugars, resulting in improved cell adhesion, but contained less axons, astrocytes, and macrophages compared to peptide-functionalized PHPMA when implanted in rat cortex or optic tract (Plant et al., 1997). PHPMA was subsequently studied in rat cortex and transected spinal cord and demonstrated angiogenesis and ingrowth of axons and glial cells (Woerly et al., 1999). To further promote axonal extension, PHPMA was next combined with fibroblasts engineered to express BDNF and/or CNTF and was inserted into cavities in the optical tract. Increased neurite outgrowth was seen in animals exposed to growth factor-expressing fibroblast hydrogels (Loh et al., 2001).

While generally unsuccessful in promoting functional regeneration, these initial studies were important because they demonstrated that biomaterials can be placed into the CNS and can be used as drug delivery vehicles. Currently, hydrogels are the most frequently used biomaterial strategy to deliver drug. There are many reviews on biomaterials, particularly hydrogels for SCI (Madigan et al., 2009; Straley et al., 2010; Aurand et al., 2012; Khaing et al., 2014; McKay and Gibert, 2014; Shrestha et al., 2014; Tam et al., 2014; Assunção-Silva et al., 2015). While many hydrogels have been tested in SCI models, we will focus on those that have been fundamental in biomaterial-drug delivery development for SCI. Later, we will discuss modifications that can be made to hydrogels for drug delivery.

Depending on the shearing properties of the hydrogel, some hydrogels can fill the injury cavity and simulate mechanical properties of CNS tissue (Assunção-Silva et al., 2015). Other hydrogels are colloidal solutions within the syringe but solidify following injection in response to changes in temperature, pH, or other stimuli (Nguyen and Lee, 2010). Agarose is an injectable, natural carbohydrate polymer explored for nervous system applications. A 2% agarose formulation containing BDNF was applied to a T10 modified over-dorsal hemisection rat model of SCI. The agarose was injected into the injury site and then cooled post-injection to solidify within the lesion (Jain et al., 2006). Despite the challenges of applying a cooling mechanism to solidify the agarose hydrogel *in situ*, the desirable characteristics of agarose (ability to inject the hydrogel and innate porosity) make it an appropriate vehicle for drug delivery for nervous system applications (Bellamkonda et al., 1995).

To circumvent the complication of cooling the hydrogel to solidify it within the lesion, the natural polysaccharide polymer HA is combined with the cellulose derivative, MC (Gupta et al., 2006; Caicco et al., 2013; Pakulska et al., 2015). HA initiates wound healing but is unable to solidify naturally, while MC reversibly crosslinks in response to increased temperatures. A HA (1 wt%) and MC (3 wt%) hydrogel was injected into a rat T2 clip compression SCI model and stiffened in response to the 37°C environment (Baumann et al., 2010). Combining HA and MC results in a hydrogel well-suited for drug delivery to the injured spinal cord via injection. HAMC hydrogels are minimally invasive, gel quickly *in situ*, and have the added benefit of sealing the dura post-lesion (Gupta et al., 2006).

Other natural hydrogels, such as the protein hydrogel fibrin, are placed (not injected) within hemi-section SCI models as they are already in the solid phase before insertion to the injury site (Wilems and Sakiyama-Elbert, 2015; **Figure 1A**). Fibrin is a blood protein involved in the coagulation cascade and is formed when the glycoprotein, fibrinogen, is cleaved by thrombin, a protease. This exposes polymerization sites, enabling fibrin monomers to polymerize into hydrogel form (Li et al., 2015). Through reduction of thrombin concentration (to create a partially solidified hydrogel) or through inclusion of a co-polymer such as alginate (that can interrupt fibrinogen chain entanglement), fibrin hydrogels may be injected into the SCI site (Straley et al., 2010; Sharp et al., 2012; des Rieux et al., 2014). Fibrin is advantageous as it is degraded naturally. Depending on the fibrinogen to thrombin ratio, fibrin also degrades rapidly (Bensaïd et al., 2003), potentially hindering axonal regeneration into the lesion site as there is no structural support.

One of the most common synthetic hydrogel materials found to be restorative in SCI is PEG. PEG is a neutral, water-soluble polymer that is known to fuse with the plasmalemma of damaged cells (Borgens and Shi, 2000; Luo et al., 2002). A study utilizing PEG assessed axonal regeneration 5 weeks following either partial or complete T hemisection injury in rat. PEG hydrogel was more effective in regenerating axons than alginate or Matrigel^®^, potentially due to the softness of PEG (Estrada et al., 2014).

### Tuning Hydrogel Drug Release

**FIGURE 2:**
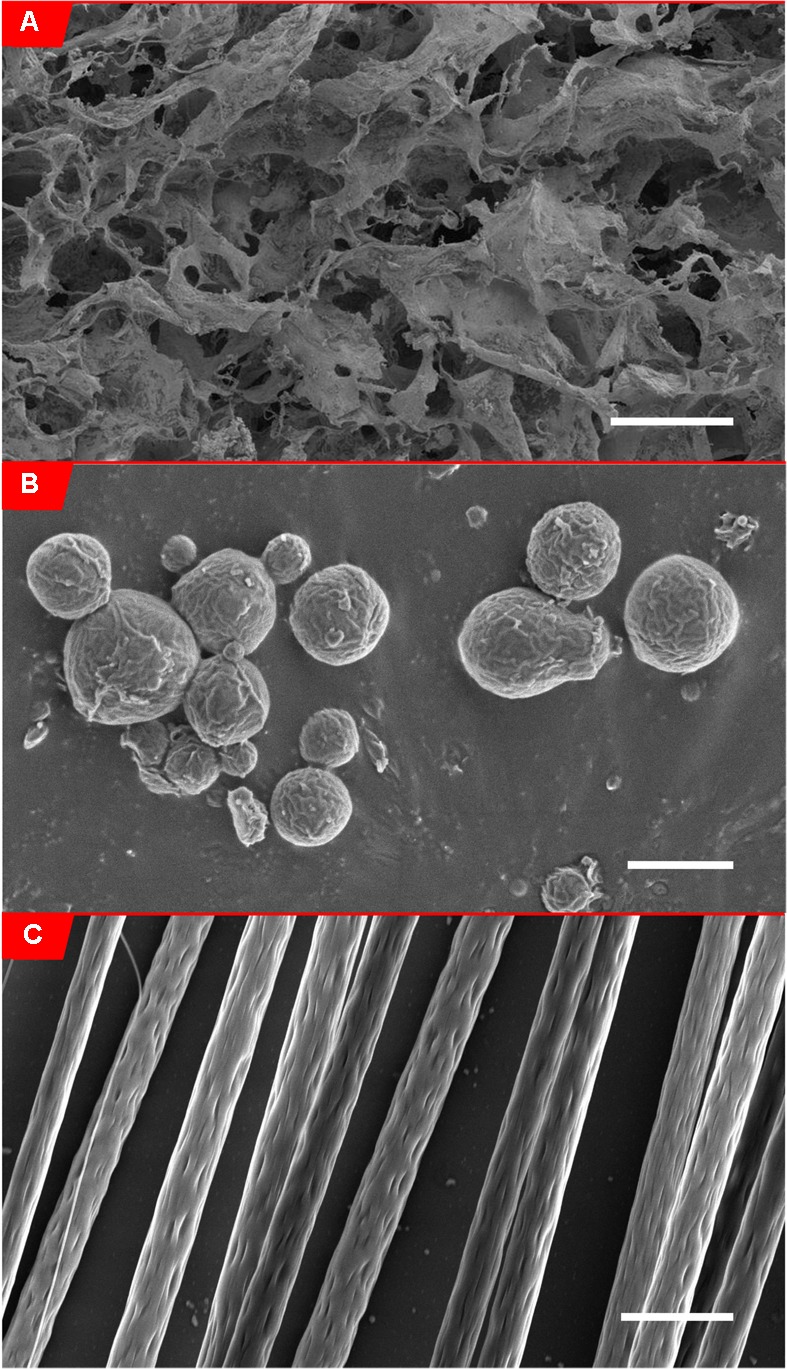
**Scanning electron micrographs of biomaterials. (A)** Scanning electron micrograph of alginate hydrogel (0.5% w/v in 0.85% NaCl solution) (McKay et al., 2014), scale bar: 200 μm. (Panel B from Figure 5 obtained from McKay et al., 2014). **(B)** Scanning electron micrograph of PLLA-coated FeO_2_ microparticles (6.6% w/w FeO_2_/PLLA containing 6.6 × 10^-4^% w/w chABC/PLLA), scale bar: 2.5 μm. **(C)** Scanning electron micrograph of aligned electrospun PLLA fibers (8% w/w in chloroform), Scale bar: 5 μm.

Hydrogels can provide a localized delivery of drugs over short periods of time (hours to days due to large hydrogel pore sizes). Hydrogels may be engineered to extend the duration of release or combined with other drug delivery vehicles that can extend the duration of release from the hydrogel. Varying the porosity of a hydrogel alters the diffusion rate of drugs from hydrogels (**Figure 2A**). Investigators are developing hydrogels with more controlled porosity to better regulate the rate of drug release (Hoare and Kohane, 2008). Increasing the polymer concentration (Bertz et al., 2013), monomer concentration, and amount of cross-linking (Kremer et al., 1994; Bertz et al., 2013) all result in hydrogels with smaller pore sizes. Increasing the polymer concentration leads to decreased drug release (Peppas et al., 1999). Similarly, increasing the level of crosslinking decreases the diffusion rate of drug. Pakulska et al. employed physical and chemical crosslinks in HAMC hydrogels and added drug-releasing PLGA NPs to further control drug delivery, providing a sustained release for more than 4 weeks with no initial burst (Pakulska et al., 2015).

Surfactants are used to improve drug solubility in hydrogel materials. Surfactant addition to hydrogel biomaterials can also influence porosity. A study of drug release from agarose hydrogels with sodium lauryl sulfate, Tween 80^®^, and Pluronic F-68^®^ demonstrated that increasing the percentage of hydrophobic surfactants (particularly Tween 80^®^ but also Pluronic F-68^®^) while simultaneously incorporating a hydrophobic drug significantly decreases pore size and overall percent porosity (Marras-Marquez et al., 2014). Like the addition of surfactants, adding co-polymers affects the porosity of hydrogels. Addition of the polysaccharide carrageenan to gelatin increases the pore size, ultimately increasing the drug release rate (Varghese et al., 2014).

Post-processing approaches are also used to tune pore size and include particle leaching, lyophilization, and gas foaming. For particle leaching, a uniformly sized solute is incorporated within the polymer solution. The polymer is solidified, and the solute is removed by leaching or dissolution in an appropriate solvent, leaving behind a uniformly porous hydrogel. Lyophilization, or freeze-drying, involves cooling of the polymer solution under vacuum, which enables sublimation of the solvent. Spaces the solvent previously occupied become pores within the polymer network. Gas foaming is achieved by incorporating a foaming agent, or compound that produces gas as it decomposes, enabling the formation of pores within the hydrogel (Annabi et al., 2010). More complex methods of hydrogel degradation control (e.g., β-elimination) can be employed to further slow the drug release and maintain hydrogel structure even after release. The release occurs via non-enzymatic drug-hydrogel self-cleavages and hydrogel crosslink self-cleavages. This release is controlled by the acidity of the proton adjacent to the cleavage sites, enabling high predictability of release rates (Ashley et al., 2013).

Affinity-based approaches have been used to slow the release of proteins. Fibrin hydrogels are modified by first crosslinking peptides to fibrin using transglutaminase, Factor XIIIa. These peptides have an affinity for heparin and covalently immobilize heparin to fibrin. Heparin has affinity for growth factors, including FGF and NGF, which ultimately slows the release of these growth factors from the fibrin hydrogel (Sakiyama-Elbert and Hubbell, 2000b). DRG were cultured within heparin-containing fibrin hydrogels that release NGF. This drug delivery system resulted in increased neurite outgrowth compared to NGF in culture medium and NGF released from a fibrin hydrogel without heparin (Sakiyama-Elbert and Hubbell, 2000a). Increasing the ratio of heparin to NT-3 resulted in increased release of NT-3 from the fibrin hydrogel. NT-3 was demonstrated to both increase DRG neurite outgrowth *in vitro* and neural sprouting in an *in vivo* rat suction ablation model (Taylor et al., 2004). Other studies also demonstrated increased neural sprouting as well as decreased GFAP expression using the heparin affinity system. However, no functional recovery was observed (Taylor and Sakiyama-Elbert, 2006; Taylor et al., 2006; Johnson et al., 2009).

Similarly, HAMC hydrogels have been modified with SH3 binding peptides to slow protein release. ChABC, an enzyme that degrades CSPGs, was recombinantly expressed as a fusion protein with SH3 protein. The peptides reversibly bind to SH3, enabling a controlled release of ChABC. The affinity strength for SH3 and SH3 binding peptides was tunable, thus enabling a tunable release rate (Pakulska et al., 2013). The system was also studied using rhFGF2, and a release over 10 days was achieved (Vulic and Shoichet, 2012). When appropriate crosslinked proteins and protein drugs are selected, affinity-based drug delivery is effective in controlling drug delivery from hydrogels. To better tune the release of drugs for SCI, these strategies could be applied based on the selected hydrogel and drug.

As described above, hydrogels possess desirable characteristics for local drug delivery to the injured spinal cord. Hydrogel injectability and ability to change morphology are important for conforming to injuries of irregular geometry. The topographical drawback of hydrogels is that they lack guidance features necessary to promote white matter tract regeneration. Future generations of hydrogels must also overcome their general inability to control the release of drug. Without additional processing, many hydrogels release drug quickly due to their highly porous network and their large pore sizes. While rapid drug release may be desirable for influencing the inflammatory response, a longer, sustained release is required if the drug’s purpose is to support axonal regeneration.

### Nano- and Micro-Sized Particles and Tubes for SCI

Particles and tubes can be fabricated to possess nano- to micro-sized geometries composed of polymer, lipid, silica, and carbon in the case of nanotubes (Masserini, 2013; Tyler et al., 2013). For CNS delivery, polymer materials are often preferred due to their biocompatibility (Patel et al., 2012). Historically, NPs have been employed to enable CNS delivery of drugs that are too large or lipid insoluble to cross the blood-brain barrier. Drugs with molecular weights below 400 g/mol and high lipophilicity have increased blood-brain barrier permeability (Levin, 1980). Unfortunately, when these lipophilic drugs are delivered systemically, they bind to hydrophobic binding pockets of plasma proteins, preventing delivery to the target site (Begley, 2004). Proteins are often incorporated onto the surface of NPs to enable targeting of specific tissues and ultimately uptake via transport methods like endocytosis (Begley, 2004; Patel et al., 2012). Kreuter et al. conducted the first study that delivered drug-containing NPs to the CNS. In this study, dalargin, a hexapeptide, was bound to the surface of poly(butyl cyanoacrylate) particles. These NPs were taken up by endothelial cells using phagocytosis, ultimately producing an analgesic effect (Kreuter et al., 1995).

For SCI injury, nano- and micro-sized particles and tubes are employed to provide a more sustained release of drugs. Unlike hydrogels, individual nano- or micro-sized drug-delivery particles or tubes are unable to fill a SCI lesion. Thus, to keep particles and tubes evenly dispersed throughout the lesion site, drug-delivery particles or tubes are placed within hydrogels (**Figure 1B**). Additionally, the hydrogel provides another barrier to the drug reaching the surrounding spinal cord tissue, lengthening the duration of release or more finely tuning the amount of drug released. One of the most common materials used for NP fabrication for SCI is PLGA. PLGA is biodegradable via hydrolysis and is FDA approved as a drug carrier (Danhier et al., 2012). Drug-free PLGA NPs were incorporated into HAMC hydrogels. This material demonstrated no significant effect on inflammation, lesion size, and functional outcome (Baumann et al., 2010). Stanwick et al. maintained a sustained release of anti-nogoA, a nogoA antagonist that prevents growth cone collapse and demyelination, from PLGA NPs within a HAMC hydrogel. Release occurred over 4 weeks while maintaining bioactivity (Stanwick et al., 2012). To target neurons, oligodendrocytes, and stem cells, sustained growth factor release has been studied *in vitro*. CNTF was included in a photoinitiation-polymerized PEG hydrogel at 20 and 30 weight percent. The hydrogel containing CNTF was used in a dual release material with NT-3-releasing PLGA microspheres. NT-3 demonstrated a linear long-term release (>60 days) compared to the burst of CNTF. CNTF release from the PEG hydrogel resulted in an increased number of neurites (Burdick et al., 2006).

Similarly, chitosan microspheres have been used to deliver FGF. Chitosan was crosslinked to heparin, which has a high binding affinity for FGF. Neural stem cell growth was enhanced when exposed to the FGF on the scaffold compared to FGF within the media (Skop et al., 2013). In another study, PLGA NPs containing PDGF-AA within a HAMC hydrogel were delivered to increase differentiation of neural progenitor cells into oligodendrocytes. PDGF-AA release from HAMC alone occurred within 2 days while PDGF-AA from NPs resulted over 21 days. Co-inclusion of PEG in the NPs further slowed the PDGF-AA release and reduced the overall amount of PDGF-AA released, potentially due to increased aggregation. With PDGF-AA, more cells were labeled for Rip, suggesting higher oligodendrocyte populations (Elliott Donaghue and Shoichet, 2015).

Several studies have placed drug-releasing particles within rodent models of SCI (Chvatal et al., 2008; Kim et al., 2009; Lee et al., 2010; Cerqueira et al., 2013; Papa et al., 2014; Ren et al., 2014; Wilems and Sakiyama-Elbert, 2015). For treatment of rat spinal cord contusion injury, Chvatal et al. incorporated MP into PLGA NPs within agarose hydrogels and found that MP was still releasing at 7 days, ultimately reducing inflammation and lesion volume at 7 days post-injury (Chvatal et al., 2008). In a combinatorial strategy, NEP1-40, a Nogo-66 receptor antagonist peptide, was delivered via PLGA microparticles, while chABC was delivered via lipid microtubes, both of which were contained within a fibrin hydrogel. Incorporation into microparticles or microtubes slowed the *in vitro* release of active chABC and NEP1-40 for up to 1 week and over 2 weeks, respectively. This ultimately decreased CSPG deposition and increased neuronal outgrowth *in vivo* (Wilems and Sakiyama-Elbert, 2015). Similarly, chABC was also delivered via a lipid microtube within an agarose hydrogel and was found to be biologically active for 2 weeks *in vitro.* Treatment with these microtubes improved locomotor function in rat T10 hemisection injury (Lee et al., 2010).

### Tuning Particle Drug Release

Particles may be fabricated to have a tunable release and to diminish burst release that often occurs during the first several hours following implantation. A likely cause of the burst release observed from particles is the retention of drug on the surface. Additionally, the rapid diffusion of drug through cracks and pores that are a product of NP fabrication or fast degradation/dissolution of polymer may also contribute to the magnitude of the burst release (Yeo and Park, 2004; Kamaly et al., 2016). Many factors affect degradation/dissolution rate of polymer particles, including hydrophobicity, crystallinity, porosity, molecular weight of polymers, co-polymer composition, and external factors including pH and ion concentration (Park, 1995; Anderson and Shive, 1997). As described in above, a primary strategy used to control the release of drugs from biomaterials for SCI is to incorporate particles within hydrogels. However, there are many strategies used for other applications that can tune the release, and those will be discussed further.

Particle size dramatically affects release rates (Wang et al., 2008). Smaller particles, having a higher surface area to volume ratio, induce faster release kinetics than larger particles (**Figure 2B**). Larger particles can hold more drug than smaller particles, and thus, may be more appropriate for strategies needing longer release. Altering different particle formulation parameters can have a major effect on particle size. Increasing the miscibility of organic solvents used to dissolve the drug and polymer in water resulted in smaller PLGA-PEG NPs, while increasing polymer concentration in solvent resulted smaller NPs (Cheng et al., 2007). Particle size is decreased by increasing the power and duration of sonication, both of which increase the energy causing the polymer droplet to break down when it is added to the aqueous phase during preparation (Budhian et al., 2007).

Studies using other model systems can also be applied to SCI drug delivery. Using a hydrogel-lipid microtube system, Meilander et al. were able to extend the release of 50% of loaded protein from 2.2 to 8.4 days. It was found that lower molecular weight proteins released faster, and loading a higher mass of protein resulted in more mass released (Meilander et al., 2001). To further tune the release rate, particles within the hydrogels can be modified. The addition of fatty acid esters to PLLA microspheres increased the release rate of cyclosporin A, and in some cases, established a biphasic response with different release rates (Urata et al., 1999). Addition of sodium chloride also decreases the burst release; however, the salt denatured NGF released from these particles (Péan et al., 1998). Thus, depending on the desired timescale of release, different particle modifications can be employed to enable an early, rapid release or delayed, extended release.

Particles can be incorporated within hydrogels to enable a localized drug release. The principal benefit of particle use within models of SCI is to extend the timeframe of release and better control the release dosage. The ability to extend a local release of drugs and better tune the dose using particles circumvents the need for sustained release using osmotic mini-pumps and the complications that often accompany their use. While particles provide a more controlled, sustained drug release than hydrogels, they also do not provide the structural support necessary to support axonal regeneration and promote functional recovery. To ultimately reestablish axonal circuits, biomaterials that provide topographical guidance for growth are necessary.

### Guidance Conduits for SCI Treatment

Fibers and conduits are polymeric, synthetic scaffolds that provide the anisotropic guidance cues that are lacking from hydrogel and particle approaches, and the main goal of this approach is to trigger white matter tract regeneration (**Figure 1C**). Successful conduit designs were initially fabricated using agarose and PLGA. Stockols et al. conducted one of the first studies implanting conduits into an *in vivo* SCI model. Multi-channel agarose conduits containing bone marrow stromal cells were engineered to release BDNF. In a rat cervical microaspiration injury model, the combination of the BDNF release and guidance conduits enabled neurite outgrowth in an organized, anisotropic manner (Stokols et al., 2006).

Many of the initial conduit studies focused on material characterization before transitioning to studying axonal extension within the channels. A study by de Ruiter et al. examined the permeability and mechanical properties of single and multiple channel PLGA conduits of varying lactic: glycolic acid ratios. The number of channels did not affect conduit permeability and flexibility. The methodology used in this study provides a protocol to assess the suitability of use *in vivo* (de Ruiter et al., 2008). Subsequently, these multi-channel conduits were loaded with Schwann cells and implanted in a rat transection SCI model. Scaffolds with smaller diameter channels (450 μm) enabled penetration of more axons per channel and resulted in a smaller fibrous rim (Krych et al., 2009). He et al. also fabricated multichannel conduits and demonstrated that increasing PLGA concentration increased the scaffold modulus and decreased the porosity. These conduits integrated into the rat T10 transected spinal cord by 8 weeks (He et al., 2009).

Poly(lactic-co-glycolic acid) scaffolds were implanted within a T10 hemisection injury site. The multichannel scaffolds resulted in decreased CSPG intensity and were positive for neural fiber growth within the channels (Yang et al., 2009). In another study, drug release from PLGA multi-channel scaffolds was modeled using fluorescein isothionate–dextran (FITC-D, 167 kD), which is an appropriate estimate for proteins of a similar molecular weight. Fluorescein isothionate–dextran burst release occurred during the first 48 h and continued to release during the next 12 weeks. When the scaffold was implanted in a rat T9 transection model, regenerating axons were seen at 1 month and were present throughout the channel (Moore et al., 2006). Later, Schwann cells and neural stem cells were seeded in these scaffolds and additional axonal regeneration was observed (Olson et al., 2009).

In place of cells, growth factors are another option to encourage axonal regeneration within the scaffold. In one study, fibroblasts exposed to BDNF released from layered PEG and poly(acrylic acid) agarose crosslinked scaffolds had increased proliferation, demonstrating that much of the bioactivity of BDNF was retained. High levels of BDNF were released during the first few days, leveling off around day 5 (Lynam et al., 2015). The combination of the conduit’s physical guidance and the growth factor’s chemical reinforcement would be a suitable treatment strategy for proliferative and chronic stages, which require cell growth and migration. Unfortunately, this treatment strategy is applicable to transection models which is less relevant than contusion injury.

While the use of guidance conduits clearly demonstrates their capability of directing axonal extension in the presence of growth factors and other molecules, the strategies place conduits within animals immediately after injury. Growth factor release immediately after injury can help protect neurons from secondary injury (Garcia et al., 2016). However, the scaffolds must also enable long-term delivery of growth factor to regenerate axons during the chronic phase of injury. Furthermore, these rigid scaffolds are employed in complete transection or hemisection models that do not recapitulate the most common form of human SCI (contusive injury). Opportunities exist to craft scaffolds capable of being implemented in contusive injury models to guide regenerating white matter tracts.

### Electrospun Fibers for SCI Treatment

Fibrous materials trigger anisotropic extension of neurites which is important for white matter tract regeneration (**Figure 1C**; Xie et al., 2010; Lee and Livingston Arinzeh, 2011; Schaub et al., 2015). Nano- to micro-sized polymer fibers are produced by electrospinning. In this process, a polymer solution is extruded into an electric field. The high voltage causes ions to be pulled to the tip of a polymer cone. The electrical forces eventually outweigh the surface tension of the polymer solution, pulling the solution into a long thin fiber (Reneker and Chun, 1996; Mu et al., 2014). As the fiber is whipping unstably in the electric field, it can be collected on a rotating mandrel spinning at high speeds to produce highly aligned scaffolds. PCL and PLLA are biodegradable polymers commonly used to fabricate electrospun fibers scaffolds for SCI. PLLA is very similar to PCL with the exception of being less hydrophobic, thus enabling a faster degradation if the molecular weight of the polymers is similar.

Electrospun fibers were first fabricated for CNS applications by Yang et al. (2004). Random PLLA fibers were electrospun and enabled neural stem cell differentiation and neurite outgrowth (Yang et al., 2004). The Ramakrishna lab later studied blends of PCL and gelatin and demonstrated increased neurite outgrowth of C17.2 nerve stem cells compared to PCL control fibers (Ghasemi-Mobarakeh et al., 2008). Currently, the primary *in vitro* culture model used to study electrospun fibers is dorsal root ganglia (DRG), which extend long cellular processes along highly aligned fibers. Schnell et al. observed greater DRG outgrowth on PCL fibers compared to PCL/collagen fibers (Schnell et al., 2007). DRG exhibited more directional neurite extension on aligned fibers than on random fibers for PCL (Xie et al., 2009), PLLA (Corey et al., 2007), and collagen nanofibers (Liu et al., 2012a) as well as PLLA microfibers (Hurtado et al., 2011). The *in vitro* work was supported by an *in vivo* study using a rat T9-10 transection model. Hurtado et al. demonstrated that conduits lined with aligned fibers promoted significantly more axonal regeneration compared to conduits with film or randomly oriented fibers (Hurtado et al., 2011). These studies all emphasize the importance of physical guidance cues for directed neurite extension.

Several studies have used fibers to deliver drugs for SCI treatment. To decrease astrocyte metabolic activity, Schaub and Gilbert incorporated 6AN into PLLA fibers. Higher amounts of 6AN were released when a higher concentration (20%) was incorporated into fibers. High and low concentrations of 6AN demonstrated approximately linear release characteristics with the high concentration exhibiting more of an initial burst release. This high concentration of 6AN resulted in less cell attachment to fibers and fewer defined neurites. Increasing concentration of 6AN resulted in lower metabolic activity of astrocytes by an MTS assay (Schaub and Gilbert, 2011).

In one study, rolipram was incorporated into an alginate layer on top of a PLLA fiber mat at 25 and 500 g/mL. Rolipram is a small molecule drug that has anti-inflammatory properties. Rolipram had a burst release during the first 18 h. In a rat cervical hemisection model, low dose rolipram scaffolds resulted in significantly lower GFAP expression and significantly higher neurofilament expression compared to untreated control animals. Ultimately, this treatment also resulted in increased open field and movement scores compared to untreated control animals (Downing et al., 2012).

A third study incorporated NT-3 and chABC onto electrospun collagen fibers. NT-3 or chABC along with heparin were crosslinked to the scaffolds. NT-3/heparin scaffolds burst released, with 72.5 % of protein eluting within the first 6 days, but continued releasing up to 28 days. DRG had similar neurite extension on NT-3 releasing scaffolds compared to soluble NT-3 treatments. Additionally, the incorporation of ChABC with heparin extended ChABC activity for at least 32 days (Liu et al., 2012b). Ultimately, this study provides an alternative for fiber-drug loading that is compatible with proteins, whose bioactivity often diminishes.

Another approach combined drug-releasing particles with fibers to promote directional extension of neurites. Fibers were coupled with NGF-releasing PLLA-coated iron oxide particles. NGF was released linearly for 6 days up to a maximum of 6 ng. The positioning of the particles enabled a localization of drug delivery of NGF. This enhanced DRG outgrowth along fibers (Zuidema et al., 2015).

### Tuning Fiber Drug Release

Fiber modifications can be made to enable a brief or sustained delivery of drugs. Altering fiber diameter and ultimately surface area can change the drug release rate (**Figure 2C**). Smaller diameter fibers have demonstrated a greater burst release, while larger fibers provide a more sustained release, likely due to lower surface area of larger fibers and greater volume of polymer to diffuse through (Chen et al., 2012). Xie and Buschle-Diller demonstrated that when fibers are used for drug delivery and a co-solvent (e.g., methanol) is incorporated, fiber diameter can be manipulated. Fiber diameter is dramatically decreased for electrospun poly(D,L-lactic acid) fibers by increasing the methanol (co-solvent):chloroform concentration. This modification increased the drug loading efficiency. However, the release was drug-dependent. For tetracycline, smaller diameter fibers (220 nM) released more drug compared to larger diameter fibers. For chlortetracycline, smaller diameter fibers released less drug, which is likely due to the insolubility of chlortetracycline at higher concentrations (Xie and Buschle-Diller, 2010). The addition of drugs alone affects the diameter as well. Electrospinning PLLA fibers with either riluzole (small molecule) or NT-3 (protein) reduced fiber diameter (Johnson et al., 2016). Ultimately, a balance needs to be achieved between fiber diameters that result in an appropriate drug release as well as fiber diameters that are the most conducive to neurite outgrowth (Wang et al., 2010). Johnson et al. discusses how drug addition affects fiber properties and how to compensate for changes in diameter, fiber alignment, density, and morphology (Johnson et al., 2016). Different polymer-drug systems require different modifications to optimize fiber properties and ultimately drug release.

The Xie and Buschle-Diller results emphasize that the solubility limit of drug in polymer is critical. Passing the solubility limit can result in crystallized drug on the surface and inside of the fibers, resulting in more of a burst release (Natu et al., 2010). Another study demonstrated a similar result. This burst was likely due to opposite polymer-drug phobicity when attempting to spin hydrophobic PCL with a hydrophilic drug. The drug crystallization on the surface was eliminated when the drug was switched to a hydrophobic drug (Seif et al., 2015). These results suggest the importance of choosing polymers that homogenize well with drugs.

Based on fiber formulation, the degradation can be tuned (Liu et al., 2008; Straley et al., 2010). Increasing fiber crystallinity reduces drug elution rate by minimizing amorphous regions that are more accessible to water and more conducive to drug effusion. For the same reason, a higher crystallinity can reduce the degradation. Crystallinity has been shown to increase during degradation because chain rearrangement is possible (Natu et al., 2013). Similarly, a less hydrophobic polymer means water can enter more easily for hydrolysis/dissolution and ultimately break down the fiber faster. Furthermore, a modeling study suggests that a matrix of aligned fibers would result in a slower drug release than randomly oriented fibers, potentially due to smaller pore sizes within the mesh of the fibers to facilitate drug diffusion (Nakielski et al., 2015). For SCI, mats of fibers must be well aligned to enable directional neurite outgrowth, and this characteristic is important for drug release. Many factors, including fiber diameter, drug/fiber solubility, fiber alignment and density, are necessary considerations when fabricating fibers scaffolds for SCI and tailoring the release to target specific events.

Fibers demonstrate promise as both guidance cues and drug-delivery scaffolds. Yet, like other biomaterials, fibers are accompanied by translational challenges. Maintaining aligned fibers upon implantation and minimizing additional injury is a difficult task. However, methods to improve their usability are being studied, such as incorporation into a hydrogel and injection into the injury (Rivet et al., 2015). At this time, additional fine tuning is necessary to maintain fiber alignment upon implantation in animals.

### Summary of Biomaterial Overview

The biomaterial approaches described above have strengths and weaknesses in their application within models of SCI (**Table 1**). Some hydrogels can be injected and fill an irregular-geometry contusive injury and are mechanically similar to the injured spinal cord. However, most hydrogels must be significantly engineered to enable extended release of drug and most do not allow for the directed regeneration of white matter tracts. Drug delivery particles or tubes can extend the release of drug but typically require a hydrogel to keep the biomaterial within the lesion cavity or to further prolong release. While hydrogels and particles/tubes do not effectively guide the regeneration of white matter tract axons, guidance conduits or fibers can effectively direct regeneration. However, fibers are generally more rigid than native spinal cord tissue and are difficult to implement within a contusive injury. Overall, opportunity exists to develop drug delivery biomaterial approaches that provide aligned topography, appropriate mechanical characteristics, and specific drug release profiles to more successfully treat SCI. The next section of the manuscript will describe specific biomaterial and drug delivery considerations for chronological timeframes after injury.

**Table 1:** Advantages and challenges of using each biomaterial type.

Material Type	Advantage	Challenge
Hydrogel	Injectable/space filling Membrane sealing	No guidance cues Burst release
Nanoparticle	Controlled/extended drug release Injectable within hydrogel	No guidance cues
Fibers/Conduits	Physical guidance cues	Requires invasive surgery

## Biomaterial Therapeutics by SCI Pathophysiology Timeline

As drug delivery agents, biomaterials have the advantage of localizing drug release, having a tunable release rate, and providing physical guidance for cells. However, if these biomaterials are not placed into the lesion at the appropriate time following injury, their benefit will likely be suboptimal. For example, a drug to target inflammation will require application to the injury site within the first 24 h (Gensel and Zhang, 2015) and be most beneficial with a burst release. As another example, a drug that degrades CSPGs should be delivered over a longer period of time (over several weeks). This treatment would be most beneficial when administered between day 1 post-injury (beginning of CSPG production) (Silver and Miller, 2004) up to 1 week post-injury (when CSPG production peaks) (Iaci et al., 2007). Alternatively, to target cells during the chronic injury stage that occurs days to months post-injury, the material should provide physical guidance cues for white matter tract regeneration while delivering factors that induce faster axonal regeneration. In the following sections, we highlight *in vivo* studies that employ biomaterials at spinal cord lesions for delivery of drugs that target specific phenomena.

### Acute Injury Pathophysiology: 0–2 h Post-injury

**FIGURE 3:**
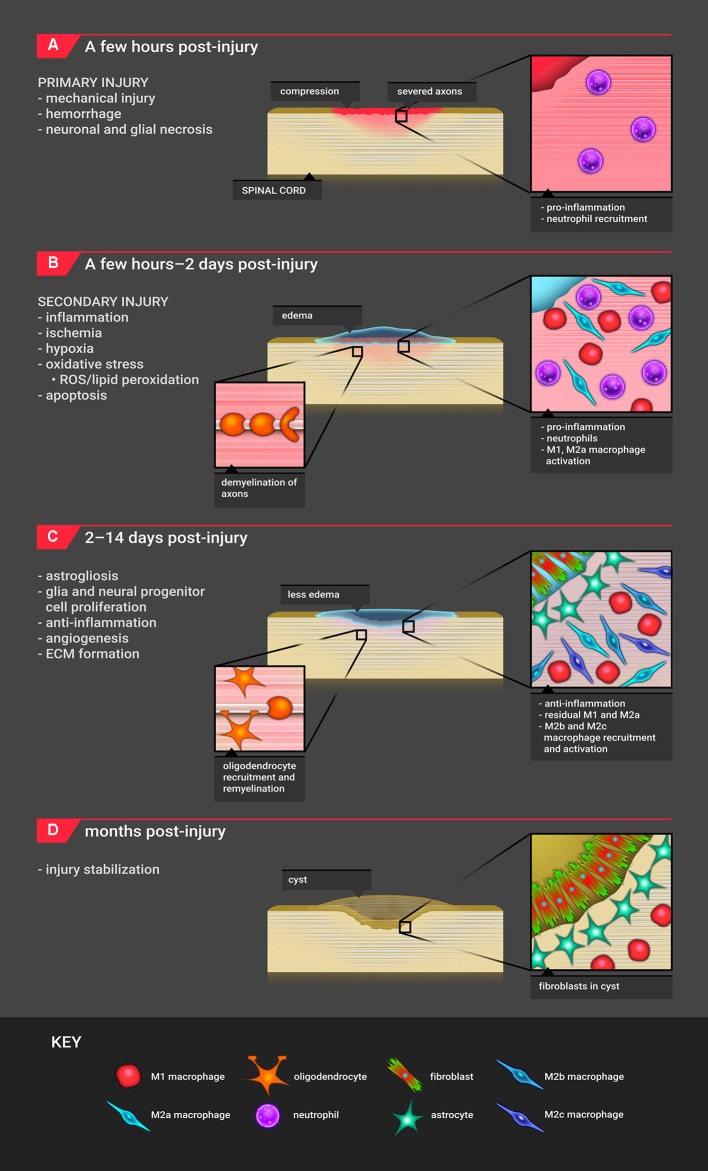
**Timeline for SCI pathophysiology. (A)** The timeline for SCI pathophysiology begins with primary mechanical injury, resulting in hemorrhage and necrosis. The major infiltrating cell type is the neutrophil. **(B)** Approximately 2 h after injury, these events initiate secondary injury processes which include edema, inflammation, oxidative stress, hypoxia/ischemia, and apoptosis. In this phase, primarily M1 as well as M2a macrophages are recruited. Demyelination of axons begins to occur. **(C)** Starting 2 days post-injury, the site shifts to an anti-inflammatory, proliferative state where M2b and M2c macrophages are recruited. Astrocytes migrate to the lesion edge, and fibroblasts produce ECM proteins, forming a scar. Remyelination of axons with oligodendrocytes occurs. The M1 macrophage response continues. **(D)** The injury site stabilizes with cyst formation; the cyst consists of fibroblasts, astrocytes, macrophages, and extracellular matrix.

The acute phase includes the immediate mechanical injury to the area (**Figure 3A**). Within 2 h post-injury, spinal cord compression initiates hemorrhaging. The hemorrhage is initially localized in the gray matter, as there is more vasculature but quickly spreads to cells in the penumbra within the first 12 h post-injury (Mautes et al., 2000; Leypold et al., 2008). By day 3, almost 75% of neuron cell bodies are lost at the epicenter of the injury site (Ward et al., 2014). The hemorrhage disrupts the blood-brain barrier and initiates an inflammatory response during the first several hours. Neutrophils begin to assemble once the blood-brain barrier is compromised, peaking at 24 h (Zhang et al., 2012), phagocytosing debris, producing ROS, and secreting pro-inflammatory cytokines (Neirinckx et al., 2014). The events during this acute phase lead to continued neuronal and oligodendrocyte necrosis which expands the lesion over time (Liu et al., 1997).

### Current Acute Injury Treatment Strategies

While hemorrhaging and necrosis are unlikely to be prevented, the initial inflammatory response can be modulated. To reduce inflammation-induced secondary injury, immediate application of a drug is required. The best biomaterial approach to enable rapid, local release of drug is to use a hydrogel. A hydrogel-NP combination may be used for extended release to target the pro-inflammatory response that extends beyond the first several hours. Hydrogels and NPs are injectable, and depending on the material composition, these biomaterials can release drug over the course of hours-days or longer. Since MP is approved clinically to target inflammation, several studies have examined the potential of biomaterials delivering MP locally, and those are described below.

Kim et al. delivered MP from PLGA NPs (MP-NP) in phosphate buffered saline as well as within an agarose hydrogel immediately after rat T9–10 hemisection injury. MP-NP delivery exhibited an initial burst release on day 1 then continued release over the next 3–4 days, which enabled continued targeting of the pro-inflammatory response. This treatment was more effective in suppressing apoptotic proteins compared to systemic delivery of MP. At 2 and 4 weeks post-injury, the lesion was about 50% smaller in MP-NP-treated animals compared to control, systemic MP, and a bolus MP. MP-NP resulted in fewer reactive inflammatory cells at the lesion and better walking ability than systemically treated and control animals (Kim et al., 2009).

Similarly, MP was applied topically to the dura of a rat T9–10 contusion injury 5 min after injury. MP was delivered from PLGA NPs interspersed within an agarose hydrogel and applied to the site. The hydrogel assisted in holding the particles within the lesion site and also acted as a barrier to better control MP release. Similar to the Kim et al. (2009) study, MP eluted via a burst release on day 1 and a sustained release was shown through day 4. This drug delivery strategy resulted in fewer macrophage and microglia, decreased calpain and inducible nitric oxide synthase intensity, and reduced lesion size (Chvatal et al., 2008).

Cerqueira et al. fabricated poly(amidoamine) and carboxymethylchitosan nanospheres loaded with MP. MP was eluted in a pH-dependent manner, with a burst release on day 1 and continued release through 2 weeks. These nanospheres were injected into T8–9 hemisection lesions immediately after injury. Hemisection lesioned rats had better BBB scores when treated with the MP-NP compared to saline, MP, or NPs (Cerqueira et al., 2013).

Methylprednisolone-conjugated chitosan NPs in phosphate-buffered saline have been used to deliver plasmid DNA immediately after a rat T9 compression injury. Using a luciferase reporter gene, Gwak et al. showed that chitosan-MP particles successfully transfected cells in the injured spinal cord. MP was conjugated to chitosan using a biodegradable ester linkage. Gwak et al. demonstrated that no plasmid was seen up to a week post-incubation, suggesting tight binding of plasmid DNA to the chitosan-MP particles. This delivery of MP resulted in lower levels of macrophages and microglia at the injury site as well as lower levels of apoptotic cells (Gwak et al., 2015).

These studies correctly target the acute window by injecting the materials immediately after injury. The materials are appropriate as many have a burst release on day 1. For even more immediate release, it may be beneficial to eliminate the use of particles. Some of the studies above saw improved functional results with hydrogel delivery of MP. To enhance this functional response, future studies may include other drugs or a combination of drugs that may provide a synergistic reduction in inflammation or combinatorial approaches with the therapies described below.

### Secondary Injury Pathophysiology: 2 h- 2 days Post-injury

Secondary injury begins 2 h after the initial injury and is most prevalent during the 1st week after injury (**Figure 3B**). Cerebrospinal fluid fills the injury cavity (Leypold et al., 2008) and is present until almost 6 months post-injury (Freund et al., 2013). Pressure build-up due to edema and vasospasm from mechanical injury restrict blood flow to the injury site, resulting in hypoxia/ischemia during the first 24 h (Sekhon and Fehlings, 2001). These events cause the activation and recruitment of microglia and macrophages within a few hours after injury (Hausmann, 2003; Hanisch and Kettenmann, 2007; Zhou et al., 2014; David et al., 2015; Gensel and Zhang, 2015). These immune cells polarize based on environmental cues; generally, M1-polarized macrophages are considered to be pro-inflammatory while M2-polarized macrophages are considered to be pro-regeneration (Gensel and Zhang, 2015). M1-polarized macrophages remove debris through phagocytosis and produce damaging ROS and pro-inflammatory cytokines (Hausmann, 2003). Oligodendrocyte cells are particularly susceptible to membrane, protein, and DNA damage from oxidative stress and glutamate excitotoxicity. Low levels of antioxidants persist months post-injury, leading to additional ROS damage (Bastani et al., 2012).

Radical species and pro-inflammatory cytokines trigger the activation of caspases, initiating the apoptosis activation cascade. White matter consisting of axons and oligodendrocytes is particularly affected (Zhang et al., 2012), due to α-amino-3-hydroxy-5-methyl-4-isoxazolepropionic acid (AMPA) receptor expression on oligodendrocytes (Park et al., 2004). Demyelination of axons proximal to the injury site is most significant 1 day post-injury and continues after re-myelination occurs (Totoiu and Keirstead, 2005). M2a macrophages facilitate tissue repair with pro-regeneration signals, beginning several days post-injury (Kigerl et al., 2009; Gensel and Zhang, 2015). The affected area attempts to reestablish neural circuitry despite a sustained opposing secondary injury response (Zhou et al., 2014; Hill, 2016). Months post-injury, macrophages remain present in areas of necrosis (Fleming et al., 2006).

### Current Secondary Injury Treatment Strategies

Similar to pathophysiology of the acute phase of SCI, treatment is most beneficial within a few hours following injury and should last at least 2 days. Thus, the use of hydrogels and NPs are most beneficial for treatment of sub-acute inflammation and secondary injury caused by these phenomena. The treatment strategy described for acute injury remains relevant as it mitigates inflammation and oxidative stress that continues during the first couple days post-injury. The use of particles enables a sustained release over days. Other key studies done to target this stage of SCI focus on mitigating apoptosis to reduce scarring and salvage neurons.

Flavopiridol is a cell cycle inhibitor used in SCI models to mitigate apoptosis. Treatment of rat T10 hemisection injury with PLGA-flavopiridol NPs 30 min after injury resulted in decreased expression of inflammatory and cell cycle genes, such as TNF-α and caspase-3 at day 3 post-injury. Histology showed fewer degenerating neurons and reactive astrocytes. Animals treated with flavopiridol NPs had better walking outcomes at day 42 post-injury compared to those treated with NPs in phosphate-buffered saline alone (Ren et al., 2014).

Similarly, dbcAMP, an analog of cAMP, was integrated into poly(propylene carbonate) fibers with the intent of reducing apoptosis, stimulating axonal growth, and decreasing astrogliosis in a spinal cord hemisection model (Xia et al., 2013). The scaffolds released dbcAMP over 8 days following an initial burst release during the first few hours. Delivery of dbcAMP from fibers resulted in more axonal outgrowth and a lessening of glial scarring than without dbcAMP *in vivo.* Unfortunately, none of the axons were able to infiltrate the glial scar to reconnect, suggesting a simultaneous need for pharmacologics that reduce inhibitory factors post-injury (Xia et al., 2013).

As seen with other studies, encapsulation of the drug dbcAMP in PLGA microspheres (over 42 days) resulted in a sustained release compared to dbcAMP in an oligo (PEG-fumarate) hydrogel alone (over 18 days). Immediate treatment with NPs containing dbcAMP resulted in less scarring in the T8–9 rat transected spinal cord than other conditions, including treatment with mesenchymal stem cells and Schwann cells. Treatment with dbcAMP NPs and mesenchymal stem cells resulted in the most improvement in locomotor function as measured by BBB testing (Rooney et al., 2011).

The inflammatory stage requires a burst treatment of anti-inflammatory drugs as described in the acute injury section. Another major component of this stage is the apoptosis of neurons and glia (Zhang et al., 2012). Appropriately, the studies above applied cell cycle inhibitors to the injury site to target cell death. The treatments were administered within 30 min post-injury, which should provide efficacy as apoptosis begins as early as 1 h post-injury (Zurita et al., 2001; Hausmann, 2003). As the release is necessary during the first 8 h in the gray matter and up to 2 weeks in the white matter (Zurita et al., 2001), an initial burst followed by a sustained release is necessary. Thus, the use of particles within a hydrogel is most appropriate for this stage.

Inflammation and apoptosis are only two phenomena that occur during this phase and are the most frequently treated in animal models. As oxidative stress and hypoxia/ischemia are also major events that occur, treatments should not overlook these phenomena. Furthermore, many studies inject or implant materials immediately after injury as the lesion is already exposed. This application is premature for some phenomena as well as less clinically translational. Future studies should consider application of drug-delivering scaffolds at more relevant time points specific to each injury phenomenon.

### Proliferation Phase Pathophysiology: 2 Days- 2 Weeks Post-injury

This stage is characterized by anti-inflammation, cell migration and proliferation, and ECM formation. At 3 days post-injury, cell populations begin to shift. M2b macrophages generate higher levels of pro-inflammatory cytokines (Mantovani et al., 2004; Sironi et al., 2006; Gensel and Zhang, 2015). M2c macrophages produce anti-fibrotic and anti-angiogenic factors to degrade the ECM through the regulation of matrix metalloproteinases (Lech and Anders, 2013). M2a and M2c macrophages are also involved in OPC recruitment and differentiation (Gensel and Zhang, 2015; **Figure 3C**). OPCs have been observed as early as 2 days post-injury, accumulating at the lesion (Ishii et al., 2001).

The reactive astrocyte response is instigated by the M1 macrophage pro-inflammatory cascade (Sofroniew, 2009; Haan et al., 2015) and mechanical strain (Cullen et al., 2007; Wanner et al., 2008) approximately 3 days post-injury. During this phase, astrocytes become hypertrophic and upregulate expression of GFAP and vimentin (Schwab and Bartholdi, 1996; Robel et al., 2011; Cregg et al., 2014). Around day 7, astrocyte proliferation peaks at the lesion edge (Sofroniew and Vinters, 2010), generating a scar (**Figure 3C**; Burda et al., 2016). Astrocytes also produce cytokines that maintain the pro-inflammatory environment and deposit ECM proteins that inhibit axonal extension. CSPGs, such as neurocan, are produced by reactive astrocytes as early as 1 day post-injury (Silver and Miller, 2004) and peak at 7 days post-injury (Iaci et al., 2007). CSPGs are potent inhibitors of axonal extension (Iaci et al., 2007; Karimi-Abdolrezaee and Billakanti, 2012). However, the astrocytic scar also maintains a necessary role during SCI recovery. Anderson et al. (2016) saw no axon regeneration when scars were prevented or ablated. It was further demonstrated that non-astrocytes were major producers of CSPGs and that astrocytes and non-astrocytes down-regulated expression of inhibitory molecules and up-regulated expression of permissive molecules 2 weeks post-injury (Anderson et al., 2016).

Approximately a week following SCI, perivascular fibroblasts migrate to the injury site (Soderblom et al., 2013) and produce ECM proteins. An astrocyte-fibroblast interface forms at the edge of the injury site, resulting in the formation of a fibroblast scar approximately 2 weeks following the injury. This scar is characterized by a dense network of collagen, particularly collagen IV and laminin in the basal lamina (Weidner et al., 1999; Klapka and Müller, 2006). While this scar shields healthy spinal cord from the injured site and re-establishes the blood brain barrier, it impairs axonal regeneration, potentially through the binding of inhibitory CSPGs (Weidner et al., 1999).

### Current Proliferation Phase Treatment Strategies

The key strategy is to first deliver drugs that reduce levels of inhibitory CSPGs then deliver growth factors to promote migration and proliferation of cells. Growth factors that promote neuron proliferation and survival, including NT-3, BDNF, and GDNF have been used extensively in addition to angiogenic growth factors that encourage wound healing. To date, the primary material types used have been hydrogels and particles; however, cell recruitment would benefit from the physical guidance cues of conduits and fibers. Application of these materials would be most beneficial between 2 and 7 days post-injury when astrocytes and oligodendrocytes are migrating into the lesion.

To first address the growth-inhibitory injury environment, inhibitory CSPGs are a major problem because they impair neuronal outgrowth, suggesting a major implication for chABC in repair (Bradbury and Carter, 2011). In a study by Lee et al., chABC was delivered topically to the lesion via lipid microtubes within an agarose hydrogel immediately after rat T10 hemisection injury. Trehalose was added to stabilize chABC and resulted in greater CSPG digestive activity for 2 weeks. The delivery of chABC alone and in combination with NGF resulted in more neurite outgrowth and greater stride length compared to other treatments (Lee et al., 2010).

One study took a combinatorial approach of applying both a competitive inhibitor of myelin degradation, NEP1-40, and chABC to make the injury site more conducive to neurite regrowth. NEP1-40 was incorporated into PLGA microparticles, and chABC was incorporated into lipid microtubes. Both were loaded into a fibrin hydrogel. Culturing dissociated DRG on myelin and CSPG inhibitory spots resulted in significantly shorter neurite processes than on areas with no inhibition. Neurite outgrowth increased with NEP1-40 and chABC treatment. Incorporation of NEP1-40 into PLGA within fibrin slowed the release and diminished the burst release compared with fibrin alone. Similar results were seen for chABC in microtubes. Neurites cultured in the presence of released NEP1-40 exhibited higher extension, suggesting bioactivity post-release. Implantation of the scaffold in a T8 hemisection injury immediately after injury resulted in less CSPG deposition and lower GFAP intensity (Wilems and Sakiyama-Elbert, 2015).

Once inhibitory factors are resolved, the next strategy would be to promote growth, proliferation, and neurite elongation using growth factors. This has been studied quite extensively for SCI. In one study, PLGA particles containing GDNF exhibited a continuous release during first 7 days. PLGA-GDNF particles were neuroprotective against glutamate-generated excitotoxicity in cortical neuronal cultures. With intraspinal injection of PLGA-GDNF particles immediately after rat T9–10 contusion injury, mice in an open field locomotor test received more than double the BBB scores than PLGA particles (Wang et al., 2008).

A collagen hydrogel containing both FGF-2 and epidermal growth factor was delivered intrathecally to rat T2 compression lesions immediately following injury. The collagen hydrogel was more effective at localizing the growth factors to the injury site than bolus injection. Epidermal growth factor permeated the collagen faster than FGF-2, releasing 67% during the first 4 days and was detected in the injury site. FGF-2 eluted slower, with 55% released over 14 days, and was trapped in the meninges. The treatment resulted in less cavitation and survival of more white matter. However, no difference was seen in the final BBB score (Jimenez Hamann et al., 2005).

Conduits and fibers are a superior choice to hydrogels and particles for this stage, as conduits and fibers promote directional guidance to cells entering the lesion. Agarose scaffolds were pre-loaded with bone marrow stromal cells genetically modified to release NT-3. These scaffolds were implanted in a rat C4 transection injury site. This treatment along with a conditioned lesion, compression of nerve to stimulate neurite outgrowth, resulted in axonal regeneration through the entire scaffold. Complete regeneration was inhibited by reactive cell layer at the interface of the lesion, emphasizing the need for treatments like chABC prior to growth factors (Gros et al., 2010).

Similarly, Yao et al. fabricated collagen conduits and inserted collagen fibers inside. The conduits were functionalized with the NT-3 gene within the polymer mixture as well as on the conduit surfaces. The NT-3 scaffold was placed in a rat T8-T10 complete transection model immediately after the lesion site was cleaned. The NT-3 gene-releasing scaffold resulted in more regenerated axons within the conduit compared to the control scaffold but failed to improve functional BBB scores (Yao et al., 2013).

The caveat with these studies is that none implement their biomaterials during the subacute window. Due to burst release, the majority of the drugs were released before the relevant time window. Johnson et al. studied a delayed implantation of materials, which is more relevant to proliferative/chronic stage pathophysiology. A T9 hemisection injury was established in rats, and the animals were sutured up for 2 weeks. They were re-opened to first remove scar tissue and then implant fibrin scaffolds. There was increased neural sprouting at 2 and 4 weeks post-implantation of fibrin (Johnson et al., 2010). For growth factor treatments to have greater functional efficacy, application during the subacute window may be more effective. Furthermore, the majority of studies have used hydrogels and NPs for delivery of growth factors. While the use of fibers and conduits poses a challenge for contusive injury implantation, these scaffolds provide the necessary physical cues to enable the migration of cells into the lesion.

### Wound Stabilization and Chronic Injury Phase: 2 Weeks- Months Post-injury

During the months post-injury, the injury site is stabilized and is continuously remodeled (**Figure 3D**). The injury site enters a chronic phase where inflammation persists. The edema initially present at the site eventually becomes a cyst, which primarily contains astrocytes, ependymal cells, fibroblasts, macrophages, and collagen (Guizar-Sahagun et al., 1994; Hackett and Lee, 2016). A subpopulation of pericytes gives rise to stromal cells, part of scar connective tissue, and are necessary for lesion closure (Göritz et al., 2011). The cyst expands over time, known as syringomyelia, causing further damage (Oyinbo, 2011). Apoptosis, hyperexcitability of cells, further demyelination of axons, cavitation, and altered neural circuitry occurs (Hulsebosch, 2002; Oyinbo, 2011). These physiological structures and events create a barrier preventing the reformation of synapses and the remyelination of neurons.

### Current Wound Stabilization and Chronic Injury Phase Treatment Strategies

It would be optimal to have Stage 3 therapeutics that continue into Stage 4 to provide growth cues until connections have been re-established. A major challenge is developing materials that sustain release of drug over weeks or months. In addition to the challenge of implanting a material into a cell and ECM-filled cyst, it is quite difficult to maintain animal models to these later time points to implant materials and study functional outcomes.

Gelain et al. fabricated PCL/PLGA guidance conduits out of electrospun fibers. Self-assembling peptides (1% w/v) were used to achieve mechanical properties similar to the spinal cord. Scaffolds in one group were filled with BDNF, CNTF, VEGF, and chABC. These scaffolds were implanted within rat T9–10 lesions after scarring was removed 4 weeks post-injury. To our knowledge, this is one of the only studies to implant a drug-releasing biomaterial during the chronic stage. Animals that were treated with the scaffolds containing growth factors had significantly increased expression of neural markers, such as βIII-tubulin. These changes were associated with significantly higher BBB scores at weeks 22 and 24 post-transplant compared to the sham control. Scaffolds without growth factors resulted in significantly greater amplitudes of descending spinal cord and cortical electrophysiological responses compared to the sham controls (Gelain et al., 2011).

While many consider the use of biomaterials to target macrophages, astrocytes, and neurons, it is also important to consider the implications of biomaterials on pericytes. The materials/drugs should support wound closure and should not obstruct tract regeneration. Due to the ease of injectable materials and ability to administer treatment immediately after injury, the acute stage of SCI has been studied most extensively. Further study of delayed implantation of fibrous scaffolds would elucidate the importance of topographical guidance for regeneration and the effect on all cell types present.

### Evaluation of *In Vivo* SCI Models Utilizing Drug-Delivering Biomaterials

Since there are many phases of SCI and each phase presents unique challenges, it is difficult to design a therapy that targets all appropriate pathophysiology. There is no current treatment that completely restores lost function as most approaches target a single phase of healing. Studies should further examine SCI pathophysiology to tease out beneficial and harmful functions of various cell types. Previously, the dogma had been that the macrophage and astrocyte responses during injury have primarily negative impacts on regeneration. However, studies have since proven that macrophage (Kigerl et al., 2009) and astrocyte (Anderson et al., 2016) responses are beneficial and imperative to recovery. To determine which physiology should be targeted and mitigated, we need to better understand cellular responses.

To date, most studies implant drug-eluting biomaterials within 30 min of injury, regardless of the type of drug being used or phenomenon being targeted. Certainly, drugs that target inflammation and oxidative stress during the first 2 days should be administered within a few hours post-injury; thus, hydrogels are a suitable approach. However, the use of hydrogels with burst release kinetics result in drug elution before relevant physiological events occur. For example, drugs that aim to reduce inhibition or promote tract regeneration would be most beneficial if administered later and for a longer duration. Based on the SCI pathophysiology timeline, material properties, and studies conducted, we propose when, how, and which type of drugs should be delivered for greatest efficacy in **Table 2**. To restore the spinal cord to a healthy state, we believe the treatment regimen would require a combinatorial approach that resolves all injury phenomena.

**Table 2:** Materials, drug classes, and time points proposed for greatest treatment efficacy at each stage to achieve maximum functional recovery.

Stage	Material Type	Drug class	Administration window (hrs post-injury)
Acute	Hydrogel, Nanoparticles	Anti-inflammatory	0–2
Inflammatory	Hydrogel, Nanoparticles	Anti-inflammatory, Anti-apoptotic, Anti-oxidant	2–72
Proliferative	Conduits, Fibers	Anti-inhibition, Growth factors	48–336
Chronic	Conduits, Fibers	Growth factors	336+

## Conclusion

Pharmacological and biomaterial approaches alone will not be able to ameliorate the damage that occurs during SCI. By taking a combinatorial approach and understanding the pathophysiology timeline, we expect to see higher efficacy in future experimental treatments of SCI. Studies that use therapeutics to address multiple injury phenomena at different time points will further elucidate the importance of this treatment strategy. Furthermore, by optimizing each material to have the appropriate release rate at a specific time, we can better target the window for each injury phenomenon. There are many strategies that can be employed to ensure the release is the most beneficial, including porosity, crystallinity, diameter, co-polymer composition, and multi-material delivery systems. Through combined pharmacological and materials approaches, we will be much closer to ameliorating SCI.

## Author Contributions

AZ and RG both planned, wrote, and reviewed the manuscript.

## Conflict of Interest Statement

The authors declare that the research was conducted in the absence of any commercial or financial relationships that could be construed as a potential conflict of interest.

## Abbreviations

6AN: 6-aminonicotinamide
ASIA: American Spinal Cord Injury Association
BBB: Basso Beattie Bresnahan
BDNF: brain-derived neurotrophic factor
chABC: chondroitinase ABC
CNS: central nervous system
CNTF: ciliary neurotrophic factor
CSPG: chondroitin sulfate proteoglycan
dbcAMP: dibutyryl cyclic adenosine monophosphate
DRG: dorsal root ganglion
ECM: extracellular matrix
EPO: erythropoietin
FGF: fibroblast growth factor
GDNF: glial-derived neurotrophic factor
GFAP: glial fibrillary acidic protein
HA: hyaluronic acid
MC: methylcellulose
MP: methylprednisolone
NF-κB: nuclear factor kappa B
NGF: nerve growth factor
NP: nanoparticle
NT-3: neurotrophin-3
OPC: oligodendrocyte precursor cell
PCL: poly(ε-caprolactone)
PDGF: platelet-derived growth factor
PEG: polyethylene glycol
PHEMA: poly(2-hydroxyethyl methacrylate)
PHPMA: poly(N-(2-hydroxypropyl) methacrylamide)
PLGA: poly(lactic-co-glycolic acid)
PLLA: poly-L-lactic acid
ROS: reactive oxygen species
SCI: Spinal cord injury
SH3: Src homology domain 3
T: thoracic
TNF-α: tumor necrosis factor alpha.
